# Suboptimal clinical response to ciprofloxacin in patients with enteric fever due to *Salmonella *spp. with reduced fluoroquinolone susceptibility: a case series

**DOI:** 10.1186/1471-2334-4-36

**Published:** 2004-09-20

**Authors:** Robert Slinger, Marc Desjardins, Anne E McCarthy, Karam Ramotar, Peter Jessamine, Christiane Guibord, Baldwin Toye

**Affiliations:** 1 Division of Infectious Disease, Children's Hospital of Eastern Ontario, 401 Smyth Rd, Ottawa, ON, Canada; 2 Division of Microbiology, The Ottawa Hospital, 501 Smyth Rd, Ottawa, ON, Canada; 3 Division of Infectious Disease, The Ottawa Hospital, 501 Smyth Rd, Ottawa, ON, Canada

## Abstract

**Background:**

*Salmonella *spp. with reduced susceptibility to fluoroquinolones have higher than usual MICs to these agents but are still considered "susceptible" by NCCLS criteria. Delayed treatment response to fluoroquinolones has been noted, especially in cases of enteric fever due to such strains. We reviewed the ciprofloxacin susceptibility and clinical outcome of our recent enteric fever cases.

**Methods:**

*Salmonella enterica *Serotype Typhi (*S*. Typhi) and Serotype Paratyphi (*S*. Paratyphi) blood culture isolates (1998–2002) were tested against nalidixic acid by disk diffusion (DD) and agar dilution (AD) and to ciprofloxacin by AD using NCCLS methods and interpretive criteria. Reduced fluoroquinolone susceptibility was defined as a ciprofloxacin MIC of 0.125–1.0 mg/L. The clinical records of patients treated with ciprofloxacin for isolates with reduced fluoroquinolone susceptibility were reviewed.

**Results:**

Seven of 21 (33%) *S*. Typhi and *S*. Paratyphi isolates had reduced susceptibility to fluoroquinolones (MIC range 0.125–0.5 mg/L). All 7 were nalidixic acid resistant by DD (no zone) and by AD (MIC 128- >512 mg/L). The other 14 isolates were nalidixic acid susceptible and fully susceptible to ciprofloxacin (MIC range 0.015–0.03 mg/L).

Five of the 7 cases were treated initially with oral ciprofloxacin. One patient remained febrile on IV ciprofloxacin until cefotaxime was added, with fever recurrence when cefotaxime was discontinued. Two continued on oral or IV ciprofloxacin alone but had prolonged fevers of 9–10 days duration, one was switched to IV beta-lactam therapy after remaining febrile for 3 days on oral/IV ciprofloxacin and one was treated successfully with oral ciprofloxacin. Four of the 5 required hospitalization.

**Conclusions:**

Our cases provide further evidence that reduced fluoroquinolone susceptibility of *S*. Typhi and *S*. Paratyphi is clinically significant. Laboratories should test extra-intestinal *Salmonella *spp. for reduced fluoroquinolone susceptibility.

## Background

*Salmonella enterica *Serotype Typhi (*S*. Typhi) and Serotype Paratyphi (*S*. Paratyphi) with reduced susceptibility to fluoroquinolones are common in India and Southeast Asia [1]. Such isolates have elevated minimum inhibitory concentrations (MICs) to ciprofloxacin and other fluoroquinolones, although they are still considered "susceptible" using current National Committee for Clinical Laboratory Standards (NCCLS) interpretive criteria [2].

Reduced susceptibility to fluoroquinolones most often arises from point mutations in the quinolone resistance determining region (QRDR) of the *gyrA *gene which encodes the A subunit of DNA gyrase. These mutations lead to resistance to nalidixic acid, a quinolone, and resistance to this agent can thus be used as an indicator of reduced fluoroquinolone susceptibility [3].

Although the mechanisms of resistance have been defined, there is still little information on the clinical significance of reduced susceptibility to fluoroquinolones in *Salmonella*. Limited published data suggest that treatment of these infections with a fluoroquinolone may result in a delay in clinical response and possibly treatment failure [2].

As a result of this clinical data, NCCLS currently recommends testing of extra-intestinal *Salmonella *isolates for nalidixic acid resistance as a marker for reduced fluoroquinolone susceptibility [4]. Others have suggested a re-evaluation of the current fluoroquinolone MIC breakpoints for *Salmonella *[2] while some recommend differentiating between "low level resistance" (MICs of 0.125 – 1.0 mg/L) and "high level resistance" (MICs >1.0 mg/L) [5].

A poor response to ciprofloxacin therapy in a patient with typhoid fever at our hospital prompted us to review retrospectively the ciprofloxacin MICs and clinical outcomes of other enteric fever cases that were treated with a fluoroquinolone.

## Methods

We tested 21 *S*. Typhi and *S*. Paratyphi blood culture isolates recovered from patients at The Ottawa Hospital and The Children's Hospital of Eastern Ontario between 1998–2002. Susceptibility testing was performed using NCCLS methods and interpretive criteria [4,6,7]. Disk Diffusion testing was performed using nalidixic acid (30 μg), ampicillin (10 μg), trimethoprim-sulfamethoxazole (1.25 μg/23.75 μg), and chloramphenicol (30 μg) disks (Oxoid, U.K.) on Mueller-Hinton agar plates (PML Microbiologicals, Mississauga, ON). MICs to nalidixic acid (Sigma-Aldrich, St-Louis, MO) and ciprofloxacin (Bayer Inc.) were determined by agar dilution using Mueller-Hinton agar (Becton Dickinson and Company, Sparks, MD). Reduced susceptibility to fluoroquinolones was defined as a ciprofloxacin MIC of 0.125–1.0 mg/L [3].

We sequenced the QRDR region of the *gyrA *gene for all isolates with reduced susceptibility to fluoroquinolones to detect nucleotide mutations. PCR amplification and DNA sequencing was performed using primers [8] and conditions previously described [9]. Sequencing was performed by the Ottawa Genome Centre DNA Sequencing Institute using the Big Dye Terminator v 3.1 method. Sequences were compared to the *gyrA *sequence of the fully susceptible *S. *Typhimurium NCTC 74 (accession number X78977, EMBL GenBank database) [8] and to 3 fully susceptible *S*. Typhi isolates.

Pulsed field gel electrophoresis (PFGE) was performed on isolates with reduced susceptibility to fluoroquinolone using *XbaI*. Medical records of patients who were treated with ciprofloxacin for infections due to isolates with reduced fluoroquinolone susceptibility were reviewed to determine clinical outcomes.

## Results

### Laboratory

Seven of 21 isolates or 33% (4 of 12 *S*. Typhi and 3 of 9 *S. *Paratyphi) were nalidixic acid resistant by disk diffusion, all with no zones of inhibition. These isolates were all nalidixic acid resistant with an MIC range of 128->512 mg/L, and demonstrated reduced susceptibility to fluoroquinolones (ciprofloxacin MIC range 0.125–0.5 mg/L). The 14 nalidixic acid susceptible isolates by disk diffusion (zone size range 20–25 mm) were also susceptible by agar dilution (MIC range 2–4 mg/L), and none had reduced susceptibility to fluoroquinolones (ciprofloxacin MIC range 0.015–0.03 mg/L). Multidrug resistance was identified in 5 of 21 isolates (24%) with resistance to ampicillin, trimethoprim-sulfamethoxazole, and chloramphenicol.

All 7 isolates with reduced susceptibility to fluoroquinolones had nucleotide point mutations in the *gyrA *gene that resulted in amino acid substitutions: 5 had a Ser83 to Phe change, one had a Ser83 to Tyr change, and one had an Asp87 to Asn change.

PFGE of the 7 resistant isolates showed 3 of 4 *S*. Typhi to have identical patterns. These 3 strains were also multidrug resistant to ampicillin, trimethoprim-sulfamethoxazole and chloramphenicol. The fourth strain, which was susceptible to ampicillin, trimethoprim-sulfamethoxazole and chloramphenicol, was a closely related strain (2 band difference). Two of 3 *S. *Paratyphi had identical PFGE patterns, with the third being possibly related (4 band difference) [10]. All 3 isolates of *S*. Paratyphi were susceptible to the other antibiotics tested.

### Clinical (Table 1- see Additional file 1)

Infections with the 7 isolates with reduced susceptibility to fluoroquinolones were all acquired in the Indian subcontinent. Five of the 7 patients with infections due to these isolates were treated initially with ciprofloxacin. The clinical and microbiological findings for these 5 are summarized in Table 1 (see Additional file 1). *S. *Typhi isolates from cases 1, 3 and 4 were also resistant to ampicillin, trimethoprim-sulfamethoxazole and chloramphenicol. The *Salmonella *isolates from the other 2 cases were susceptible to these antibiotics. Of note, 3 of 4 patients with *S*. Typhi had fever durations well in excess of the expected 95% upper confidence limit of 3.9 days found in a pooled analysis of multiple studies. These studies involved patients with predominantly nalidixic acid susceptible *S*. Typhi who were treated with fluoroquinolones [1].

The patient infected with *S*. Paratyphi with reduced susceptibility to fluoroquinolone was judged to be failing ciprofloxacin, since there was no clinical improvement after 3 days of treatment. Although fever of this duration is common, patients with enteric fever given fluoroquinolones may show some clinical improvement by this time. Three days with no amelioration of symptoms has been used as indicator of a poor response to therapy in some studies [11,12]. Thus, this patient possibly may have had a poor response to therapy due to the elevated ciprofloxacin MIC.

None of the 5 had a recurrence of bacteremia after antibiotics were discontinued, and none died.

## Discussion

Fluoroquinolones have been considered the treatment of choice for enteric fever. As noted, a mean fever clearance time of only 3.9 days (for predominantly nalidixic acid-susceptible strains) has been reported, which is shorter than for other agents [1]. Calculated clinical and microbiologic failure rates of 2.1% and 0.4%, respectively, are also low in comparison to other antibiotics [1]. Relapse rates (1.2%) and fecal carriage rates (1.5%) have also been lower than for the traditional drugs, trimethoprim-sulfamethoxazole and chloramphenicol.

However, reduced susceptibility to fluoroquinolones jeopardises the usefulness of these agents. It is now seen frequently in *S*. Typhi and *S*. Paratyphi isolates acquired in the Indian subcontinent and Southeast Asia [1], where common clones appear to be circulating. In fact, the common PFGE profile of 3 of our *S*. Typhi isolates with reduced susceptibility to fluoroquinolones appears to be the same as that described for *S*. Typhi strains from India, Pakistan, Bangladesh, and Tajikistan [13]. The *gyrA *point mutations in our isolates have also been previously described in isolates from these countries [1,3].

Several lines of evidence support the clinical significance of elevated fluoroquinolone MICs in *Salmonella *spp., including case reports, animal experiments, and pharmacodynamic modelling [2]. In a report of patients treated with short courses of oral ofloxacin [11], the median time to fever clearance was greater for patients infected with nalidixic acid resistant *S*. Typhi than for those with nalidixic acid susceptible strains. In addition, one third of nalidixic acid resistant infections required re-treatment vs. 0.8% of infections due to susceptible strains. The authors concluded that short courses (less than 5 days) of oral fluoroquinolone therapy should not be used for treating nalidixic acid resistant isolates. Our small series adds to the evidence that there is a delayed clinical response to fluoroquinolone therapy in this setting, and demonstrates that fever may be prolonged even with long courses of fluoroquinolone therapy, and even when given parenterally in some cases.

Until results from randomized controlled trials of enteric fever due to strains with reduced susceptibility to fluoroquinolones are available, the best treatment regimen is uncertain. Current options include higher dose and longer duration of fluoroquinolones, 3rd generation cephalosporins and azithromycin, either alone or in combination [1]. If susceptible, agents such as ampicillin, trimethoprim-sulfamethoxazole, and chloramphenicol may also be considered, but rates of resistance to these agents are generally too high to recommend as first-line empiric therapies [1]. In our series, resistance to all these agents was high among the isolates with reduced fluoroquinolone susceptibility (3 of 7 or 43%).

When fluoroquinolones are given, it is important to bear in mind that two pharmacokinetic/pharmacodynamic ratios are predictive of successful treatment with these agents [2]. These are the ratio of peak serum antimicrobial level to the MIC, and the ratio of the 24-hour area under the serum concentration-versus-time curve to the MIC (AUC/MIC). Higher dosing will maximize these ratios, and thus when oral ciprofloxacin is given for suspected enteric fever, use of 750 mg rather than 500 mg bid seems logical.

It is important that microbiology laboratories have procedures to detect *Salmonella *strains that have reduced susceptibility to fluoroquinolones. The current NCCLS guidelines (2004) recommend that all extra-intestinal isolates of *Salmonella *be tested for resistance to nalidixic acid in order to detect reduced fluoroquinolone susceptibility [4]. In addition, it is recommended that physicians be informed that isolates that test fluoroquinolone "susceptible" but nalidixic acid resistant may not be eradicated with fluoroquinolone therapy.

There have also been suggestions to change the NCCLS breakpoints to reflect the risk of treatment failure of *Salmonella *spp. with reduced susceptibility to fluoroquinolones [1]. This is clearly a difficult decision, since fluoroquinolones have previously been the most active antibiotic class for treatment of enteric fever. Some patients with these isolates may do poorly on short courses and lower doses of fluoroquinolones but potentially could respond adequately to higher doses and longer durations of treatment. Rather than reclassifying all isolates with reduced susceptibility to fluoroquinolones as being resistant to ciprofloxacin, others have recommended differentiating between low-level (MIC of 0.125 – 1.0 mg/L) and high-level (MIC >1.0 mg/L) ciprofloxacin resistance based on the MIC range [5].

It is not completely clear at present which of these is the optimal approach for laboratory detection of *Salmonella *spp. with reduced fluoroquinolone susceptibility. Not all laboratories routinely perform MICs and many rely on commercially-available automated susceptibility methods that cannot currently detect reduced fluoroquinolone susceptibility. The current NCCLS recommendations to test for nalidixic resistance are easy to implement for routine testing but may have reduced specificity [3]. Laboratories need to decide which approach is best for their workflow and available resources.

## Conclusions

Given the accumulating evidence, including our own clinical experience, enteric fever due to *Salmonella *spp. with reduced susceptibility to fluoroquinolones is clinically significant. It is important that laboratories test *S. *Typhi and *S*. Paratyphi, as well as other extra-intestinal Salmonella isolates, for reduced susceptibility to fluoroquinolones.

## Competing interests

None declared.

## Authors' contributions

RS: participated in the design of study and authored first draft; MD: conceived study, coordinated susceptibility testing, and revised manuscript; AM: conceived study and reviewed clinical data; KR: participated in study design and coordinated pulsed field gel analysis; PJ: reviewed clinical data and helped coordinate susceptibilty testing; CB: carried out molecular genetic studies and participated in sequence alignment; BT: conceived study and supervised study, revised manuscript

All authors read and approved of the final manuscript.

## Pre-publication history

The pre-publication history for this paper can be accessed here:



## Supplementary Material

Additional File 1 Clinical and microbiological information for patients treated with ciprofloxacin for *S. *Typhi or *S. *Paratyphi isolates with reduced fluoroquinolone susceptibility provides information regarding clinical course for patients with isolates with reduced fluoroquinolone susceptibility as well as antibiotic susceptibility and gene mutation results for these isolates Click here for file
