# Supplementary material for: Suboptimal clinical response to ciprofloxacin in patients with enteric fever due to Salmonella spp. with reduced fluoroquinolone susceptibility: a case series
Source: BMC Infect Dis. 2004 Sep 20;4:36. doi: 10.1186/1471-2334-4-36 (PMC521077; doi:10.1186/1471-2334-4-36)
Supplement: Additional File 1 — Clinical and microbiological information for patients treated with ciprofloxacin for S. Typhi or S. Paratyphi isolates with reduced fluoroquinolone susceptibility provides information regarding clinical course for patients with isolates with reduced fluoroquinolone susceptibility as well as antibiotic susceptibility and gene mutation results for these isolates [file 1471-2334-4-36-S1.doc]

**Table 1. Clinical and microbiological information for patients treated with ciprofloxacin for *S.* Typhi or *S.* Paratyphi isolates with reduced fluoroquinolone susceptibility**

**Isolate**

**Nucleotide mutation**

**(position)**

**Amino acid mutation**

**(codon)**

**Cipro MIC**

**(mg/L)**

**NA MIC**

**(mg/L)**

**NA zone**

**Disk diffusion**

**(mm)**

**NA susceptibility**

**135**

**146**

**83**

**87**

*Salmonella* serotype Typhi 1 (index)

A

G

Tyr

Asp

0.25

256

6

R

*Salmonella* serotype Typhi 2

C

A

Ser

Asn

0.125

128

6

R

*Salmonella* serotype Typhi 3

T

G

Phe

Asp

0.25

256

6

R

*Salmonella* serotype Typhi 4

T

G

Phe

Asp

0.25

256

6

R

*Salmonella* serotype ParaTyphiA 1

T

G

Phe

Asp

0.5

>512

6

R

*Salmonella* serotype ParaTyphiA 2

T

G

Phe

Asp

0.5

>512

6

R

*Salmonella* serotype ParaTyphiA 3

T

G

Phe

Asp

0.5

>512

6

R

NAS *Salmonella* serotype Typhi C1

C

G

Ser

Asp

0.015

2

22

S

NAS *Salmonella* serotype Typhi C2

C

G

Ser

Asp

0.015

4

22

S

NAS *Salmonella* serotype Typhi C3

C

G

Ser

Asp

0.015

2

20

S

**Isolate**

**Nucleotide mutation**

**(position)**

**Amino acid mutation**

**(codon)**

**Cipro MIC**

**(mg/L)**

**NA MIC**

**(mg/L)**

**NA zone**

**Disk diffusion**

**(mm)**

**NA susceptibility**

**135**

**146**

**83**

**87**

*Salmonella* serotype Typhi 1 (index)

A

G

Tyr

Asp

0.25

256

6

R

*Salmonella* serotype Typhi 2

C

A

Ser

Asn

0.125

128

6

R

*Salmonella* serotype Typhi 3

T

G

Phe

Asp

0.25

256

6

R

*Salmonella* serotype Typhi 4

T

G

Phe

Asp

0.25

256

6

R

*Salmonella* serotype ParaTyphiA 1

T

G

Phe

Asp

0.5

>512

6

R

*Salmonella* serotype ParaTyphiA 2

T

G

Phe

Asp

0.5

>512

6

R

*Salmonella* serotype ParaTyphiA 3

T

G

Phe

Asp

0.5

>512

6

R

NAS *Salmonella* serotype Typhi C1

C

G

Ser

Asp

0.015

2

22

S

NAS *Salmonella* serotype Typhi C2

C

G

Ser

Asp

0.015

4

22

S

NAS *Salmonella* serotype Typhi C3

C

G

Ser

Asp

0.015

2

20

S

| **Case** | **Isolate** | **Country of**  **Acquisition** | **Initial**  **CIP**  **therapy** | **ADM** | **Hospital Course** | **Fever**  **Duration**  **on CIP**  **(days)** | **Antibiotic**  **Duration**  **(days)** | **Hospital**  **Stay**  **(days)** | **CIP MIC**  **(mg/L)** | **NA MIC**  **(mg/L)** | **Amino**  **Acid** Change |
| --- | --- | --- | --- | --- | --- | --- | --- | --- | --- | --- | --- |
| 1 | ST | Bangladesh | 500 PO  bid x 2 d | Y | Still bacteremic on adm., febrile x 3 d on IV CIP so CRO added. Defervesced and was switched back to PO CIP. Fever returned and CRO re-started, defervesced. | 9 | 21 | 11 | 0.25 | 256 | Ser83 to Tyr |
| 2 | ST | India | 750 PO  bid | N | None | NR | 14 | None | 0.125 | 128 | Asp87 to Asn |
| 3 | ST | India | 750 PO  bid x 3 d | Y | Remained febrile for 7 d on IV CIP. | 10 | 10 | 8 | 0.25 | 256 | Ser83 to Phe |
| 4 | ST | India | PO bid  x 2 d,  dose NR | Y | Still bacteremic on adm., remained febrile for 7 d on 750 PO bid, defervesced when IV CIP begun. | 9 | 18 | 10 | 0.25 | 256 | Ser83 to Phe |
| 5 | SP | India | PO bid  x 2 d,  dose NR | Y | IV CIP x 1d, remained febrile and bacteremic, was then switched to IV beta-lactam. | 3 | 10 | 8 | 0.5 | >512 | Ser83 to Phe |

CIP= ciprofloxacin, CRO= ceftriaxone, ADM= admission to hospital, d= days, Ser=serine, Phe=phenylalanine, Tyr=tyrosine, Asp=aspartic acid, Asn=asparagine ; ST = *S*. Typhi, SP= *S*. Paratyphi, NR= not recorded
